# Perineoplasty for anal incontinence after obstetric anal sphincter injury repair: a case report

**DOI:** 10.1186/s40792-024-01917-7

**Published:** 2024-05-10

**Authors:** Masatsugu Kojima, Toru Miyake, Soichiro Tani, Sachiko Sakai, Yusuke Nishina, Sachiko Kaida, Katsushi Takebayashi, Hiromitsu Maehira, Haruki Mori, Reiko Otake, Takashi Matsunaga, Hajime Ishikawa, Tomoharu Shimizu, Masaji Tani

**Affiliations:** 1 Department of Surgery, Shiga University of Medical Science, Tsukinowa-cho, Seta, Otsu, Shiga 520-2192 Japan; 2 Medical Safety Section, Shiga University of Medical Science Hospital, Tsukinowa-cho, Seta, Otsu, Shiga 520-2192 Japan

**Keywords:** Obstetric anal sphincter injury, OASIS, Anal incontinence, Fecal incontinence, Perineal laceration, Perineal body, Bulbospongiosus muscle

## Abstract

**Background:**

The rectal and vaginal walls are typically sutured if severe perineal lacerations with rectal mucosal damage occur during vaginal delivery. In case of anal incontinence after the repair, re-suturing of the anal sphincter muscle is standard procedure. However, this procedure may not result in sufficient improvement of function.

**Case presentation:**

A 41-year-old woman underwent suture repair of the vaginal and rectal walls for fourth-degree perineal laceration at delivery. She was referred to our department after complaining of flatus and fecal incontinence. Her Wexner score was 15 points. Examination revealed decreased anal tonus and weak contractions on the ventral side. We diagnosed anal incontinence due to sphincter dysfunction after repair of a perineal laceration at delivery. We subsequently performed sphincter re-suturing with perineoplasty to restructure the perineal body by suturing the fascia located lateral to the perineal body and running in a ventral–dorsal direction, which filled the space between the anus and vagina and increased anal tonus. One month after surgery, the symptoms of anal incontinence disappeared (the Wexner score lowered to 0 points), and the anorectal manometry values increased compared to the preoperative values. According to recent reports on the anatomy of the female perineal region, bulbospongiosus muscle in women does not move toward the midline to attach to the perineal body, as has been previously believed. Instead, it attaches to the ipsilateral surface of the external anal sphincter. We consider the fascia lateral to the perineal body to be the fascia of the bulbospongiosus muscle.

**Conclusions:**

In a case of postpartum anal incontinence due to sphincter dysfunction after repair of severe perineal laceration, perineoplasty with re-suturing an anal sphincter muscle resulted in improvement in anal sphincter function. Compared to conventional simple suture repair of the rectal wall only, this surgical technique may improve sphincter function to a greater degree.

## Background

Perineal lacerations are common complications of vaginal delivery, occurring in 85% of cases [1]. The severity of perineal lacerations is determined based on the depth of the laceration. Third- and fourth-degree lacerations are the most severe, reaching the anal sphincter muscle and anal mucosa, respectively [1, 2]. For such serious injuries, the rectal wall is usually sutured at the time of delivery, along with the vagina [3]. However, third- and fourth-degree perineal lacerations are known to be a risk factor of postpartum anal incontinence, because repair may not lead to adequate recovery of anal sphincter function [4]. In case of anal incontinence due to sphincter dysfunction, re-suture the muscle is standard procedure, but dysfunction sometimes persists after surgery, which worsens quality of life [5]. Here, we report the case of anal incontinence after a fourth-degree perineal laceration repair at delivery, in which we reformed the perineal body by suturing the fascia located lateral to the perineal body in a ventral–dorsal direction, in addition to re-suturing repair of anal sphincter muscle, which led to improvement in anal sphincter function. This surgical procedure is versatile and is expected to improve sphincter function compared to conventional suture repair of anal sphincter muscle only [6].

## Case presentation

**Fig. 1:**
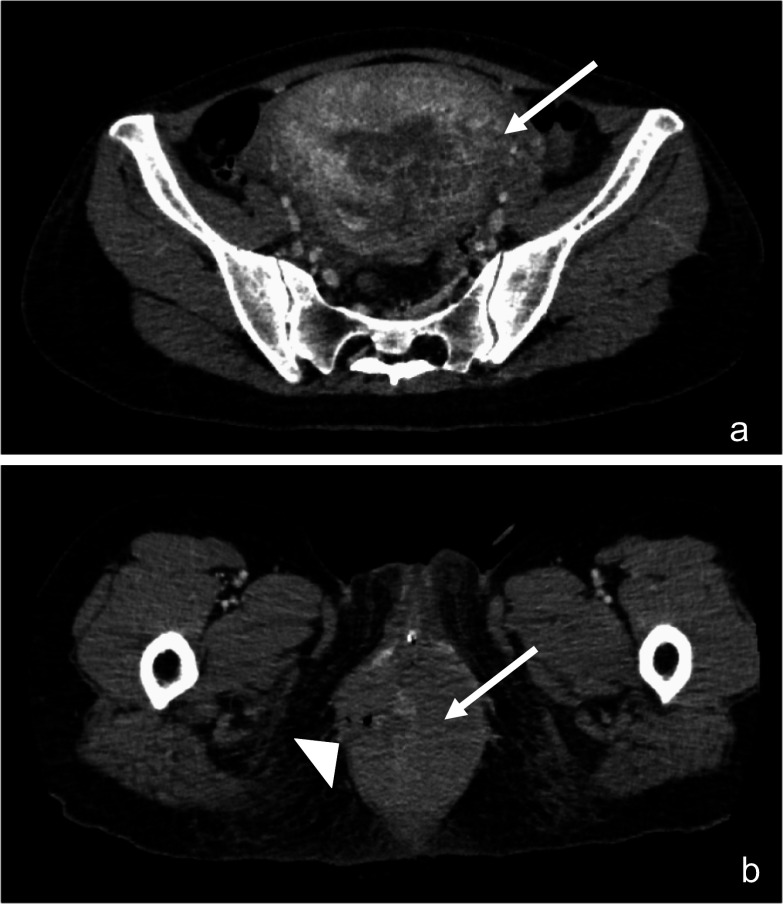
**a** A CT scan at the time of perineal laceration at delivery revealed a large amount of hematoma from the uterine cavity to the vagina (arrow). **b** A hematoma was also observed around the anus and rectum (arrowhead)

**Fig. 2:**
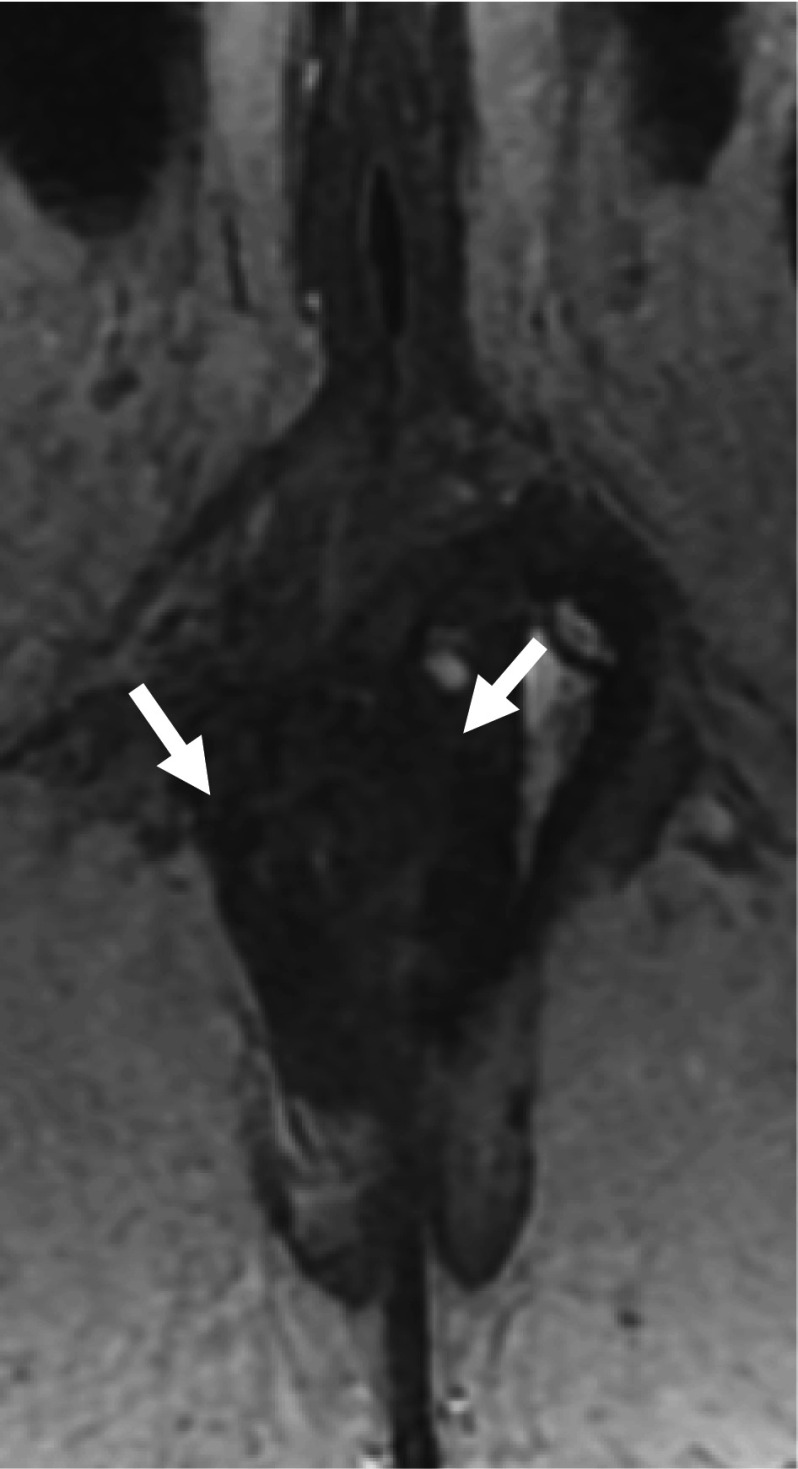
T2-weighted MRI images showed unclear continuity of the anal sphincter muscle on the ventral side. Arrows indicate the edge where the anal sphincter can be firmly recognized

A 41-year-old woman with no past medical history underwent emergent hematoma removal and suture repair of the vaginal and rectal wall for fourth-degree perineal laceration during the first vaginal delivery (Fig. 1). She was referred to our department three weeks after delivery because she had flatus and fecal incontinence (involuntary loss of liquid feces). The Wexner score was 15 points: solid stool 0, liquid stool 3,Gas 4, wear pad 4, lifestyle altered 4. Anorectal examinations revealed decreased anal tonus and weak contractions on the ventral side and no perianal fistula formation. The anorectal manometry showed a maximum resting pressure (MRP) of 7 cmH_2_O and a maximum squeeze pressure (MSP) of 35 cmH_2_O, which were lower than standard values (MRP: 92.6 ± 27.8 cmH_2_O, MSP: 218.1 ± 76.0 cmH_2_O) [7] and consistent with the anal findings. Magnetic resonance imaging (MRI) of the pelvis showed unclear continuity of the anal sphincter muscle on the ventral side, suggesting damage to the anal sphincter muscle (Fig. 2). We diagnosed anal incontinence due to anal sphincter dysfunction after repair of a fourth-degree perineal laceration at delivery. The patient was followed up until three months after delivery, but anal sphincter dysfunction had not improved. Therefore, we planned to undergo perineoplasty with sphincter re-suturing.

**Fig. 3:**
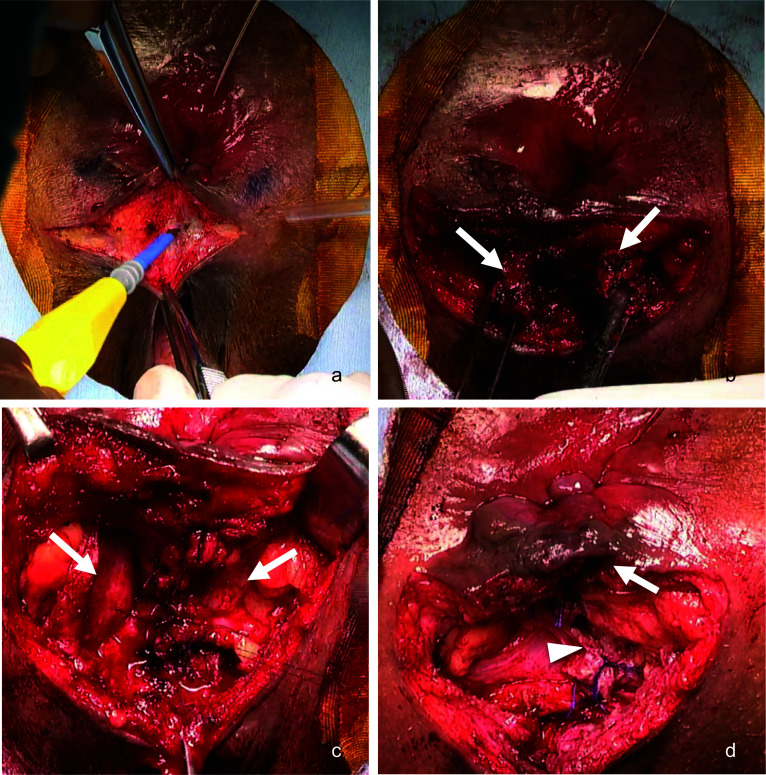
**a** The surgery was performed with the patient in the jackknife position. A transverse incision was made between the anus and vagina in the perineum, and the scar tissue from the previous surgery was deeply dissected. **b** Unrepaired anal sphincter muscles were identified bilaterally and scar tissue between them was removed, and the anal sphincter muscle was repaired with end-to-end sutures. **c** The fascia that existed on both lateral sides to the perineal body, which ran in a ventral–dorsal direction, was identified. **d** To restructure a perineal body, suturing these lateral fascias in center filled the space between the anus and vagina and increased the tonus of the anus. The force of tightening was adjusted by digital rectal examination. The arrow indicates the anal sphincter repaired by suturing end to end. The arrowhead indicates the lateral fascias sutured in the center

The surgery was performed with the patient in the jackknife position under general anesthesia. The surgical time was 130 min, and the blood loss was 136 g. A transverse incision was made between the anus and vagina in the perineum, and the scar tissue from the previous surgery was deeply dissected (Fig. 3a). Unrepaired anal sphincter muscles were identified bilaterally, the scar tissue between them was removed, and the anal sphincter muscle was repaired with end-to-end suture (Fig. 3b). The fascia lateral to the perineal body, which ran in a ventral–dorsal direction, was identified (Fig. 3c). To restructure a perineal body, suturing these lateral fascias with 3-0 absorbable threads in the center filled the space between the anus and vagina and increased the tonus of the anus. Meanwhile, the force of tightening was adjusted by digital rectal examination (Fig. 3d).

Flatulence was confirmed on the first postoperative day, but the flatus incontinence had disappeared. The patient defecated on the third postoperative day, and her fecal incontinence had improved. The patient was discharged on the fourth postoperative day without any complications. One month after the surgery, she had no recurrence of incontinence symptoms, and the Wexner score decreased to 0. Furthermore, an anorectal manometry revealed an increase in the MRP from 7 to 31 cmH_2_O and an increase in the MSP from 35 to 56 cmH_2_O. No symptom relapse was observed at 1 year and 11 months postoperatively.

## Discussion

We report a case of perineoplasty combined with anal sphincter re-suturing cured postpartum anal incontinence after fourth-degree perineal laceration repair.

Anal sphincter injuries due to perineal laceration, also called obstetric anal sphincter injuries (OASIS), occurs from 0.1% to 10.9% [8, 9]. Risk factors for OASIS include instrumental delivery with forceps, maternal age ≥35 years, and birth weight ≥4 kg [8]. When severe OASIS occurs, immediate repair is the standard of care. However, the incidence of anal incontinence following OASIS repair is high, reported to be 30% at 3 months postpartum with 4th degree perineal lacerations [5, 10].

In the treatment of OASIS, overlap and end-to-end anastomosis are two procedures for suture repair of anal sphincter muscle. A meta-analysis demonstrated that the overlap method could yield better results regarding fecal urgency and incontinence [11]. However, one or more anal incontinence symptoms (fecal urgency, flatus incontinence, fecal incontinence, alteration in fecal continence) remained after both types of procedures in approximately 20% of patients six months after surgery [11]. In our case, we considered end-to-end suturing was performed at the time of delivery properly, but anal sphincter muscle function was not sufficiently recovered. We did not believe that it would be possible to improve sphincter function only by repeating the same procedure (simple sutures), so we decided to combine re-suturing of anal sphincter muscle with perineoplasty.

Sacral neuromodulation (SNM) is another treatment for anal incontinence caused by anal sphincter injury, and some reports have recently pointed out its effectiveness [12]. Since surgery is necessary for patients who do not respond to SNM, we believe it is still important to improve the surgical procedure for OASIS. In addition, the evidence seems to be not yet sufficient for long term implantation of SNM in the body, when considering OASIS-related anal incontinence occurs mainly in young postpartum women.

Perineoplasty is the reconstruction of the perineal body by suturing the strong bilateral fascia [6]. This procedure was devised and developed mainly for anal sphincter dysfunction and rectovaginal fistula caused by perineal laceration at delivery [6, 13, 14]. However, it is only performed in a small number of hospitals, and it is not well-recognized in other hospitals. This procedure tightens the anal sphincter muscle from the outside more caudally, which strengthens the tonus to a higher degree than the anal sphincter muscle suture alone, resulting in greatly improved sphincter function. Levatorplasty by suturing the anal levator muscle is one option, but the anal levator muscle is deeper, and perineoplasty, which sutures the bulbospongiosus muscle just lateral to the sphincter, is considered more effective in improving function of the anal sphincter muscle function.

**Fig. 4:**
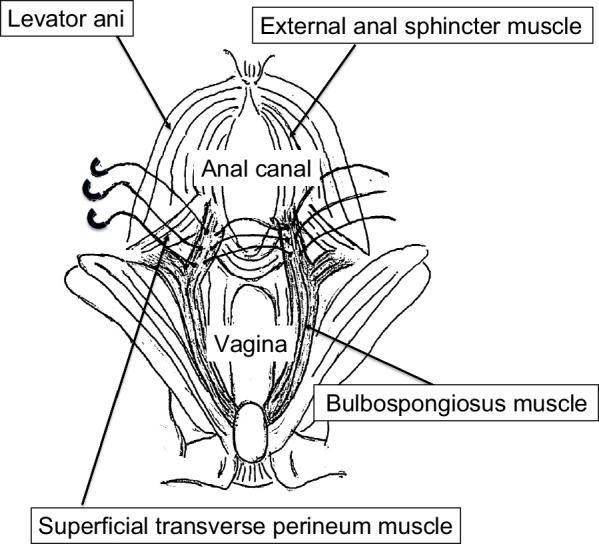
The inferior aspect of the pelvic outlet muscles in women. The bulbospongiosus muscle does not move toward the midline but adjoins the lateral surface of the external anal sphincter muscle to form a lateral connection. (Modified from Baramee et al. 2020 and Muro et al. 2023 [12, 13])

In the past, the anatomical details of the perineal body were not well understood, and there was uncertainty about the fascia on the lateral side of the perineal body. However, in recent years, detailed reports on the anatomy of the female perineal region were published that bulbospongiosus muscle in women does not move toward the midline to attach to the perineal body, as was previously believed, but instead attaches to the ipsilateral surface of the external anal sphincter (Fig. 4) [15, 16]. The running of the bulbospongiosus muscle that they describe corresponds exactly to the running of the fascia lateral to the perineum that we recognized during surgery; therefore, we believe that the bulbospongiosus muscles would be sutured during perineoplasty. The bulbospongiosus muscle is a stiff muscle, and suturing it around the anal sphincter muscle to increase its physical tension could improve anal sphincter function.

## Conclusions

When repair of a severe perineal laceration leads to refractory anal incontinence due to anal sphincter dysfunction, anal incontinence may be significantly improved by re-suturing an anal sphincter muscle and reforming the perineal body. This may be accomplished by combining the fascias lateral to the perineal body (we consider the fascia to be that of the bulbospongiosus muscles). This surgical procedure could improve sphincter function to a higher degree than conventional anal sphincter suture repair alone.

## Data Availability

The datasets supporting the conclusions of this article are included within the article.

## Abbreviations

CT: Computed tomography
MRI: Magnetic resonance imaging
MRP: Maximum resting pressure
MSP: Maximum squeeze pressure
OASIS: Obstetric anal sphincter injuries
SNM: Sacral neuromodulation
